# A Comprehensive Review of Acute Coronary Syndrome and Bypass Surgery: Recent Advances, Timing, and Indicative Considerations

**DOI:** 10.3390/jcm15020560

**Published:** 2026-01-09

**Authors:** Lőrinc Holczer, László Hejjel, István Szokodi, Attila Kónyi

**Affiliations:** 1 Heart Institute, Medical School, University of Pécs, 7624 Pecs, Hungary; hejjel.laszlo@pte.hu (L.H.); szokodi.istvan@pte.hu (I.S.); konyi.attila@pte.hu (A.K.); 2 Szentágothai Research Center, University of Pécs, 7624 Pecs, Hungary

**Keywords:** acute coronary syndrome, coronary artery bypass grafting, percutaneous coronary intervention, immediate versus staged coronary revascularization, hybrid coronary revascularization

## Abstract

Background: Acute coronary syndrome (ACS) continues to be a major contributor to morbidity and mortality worldwide. While percutaneous coronary interventions (PCIs) have significantly evolved, coronary artery bypass grafting (CABG) has retained a role in emergency revascularization. Nevertheless, ongoing debate persists about how to select candidates for surgery, when to operate, and which surgical techniques offer the greatest safety and efficacy. Methods: A comprehensive literature search was conducted, yielding 2302 records, of which 25 studies met predefined screening criteria and were included for detailed analysis. Given that timing remains one of the most controversial issues in the management of ACS, our primary aim was to determine the optimal timing for CABG in this patient population. Additionally, we examined how preoperative antiplatelet therapy and the presence of cardiogenic shock influence clinical outcomes, and what revascularization strategy may be most appropriate for these patients. Results: Of the 2302 initially identified studies, 25 were selected for a detailed analysis, supplemented by 28 additional key references. Among the included studies, 17 focused primarily on the effects of surgical timing and 8 on comparisons between the outcomes of CABG and PCI. The analysis comprised 15 database or multicentre retrospective cohort studies, 8 single-centre retrospective studies, and 2 prospective investigations. Conclusion and limitations: Although the topic of non-elective coronary surgery has been with us for several decades, a number of inherent biases hinder thorough statistical investigation in this complex population. Although a number of contradictory findings hinder drawing simple conclusions, being reluctant to perform early surgery solely based on poorer unfiltered outcomes might miss a point.

## 1. Introduction

Acute coronary syndrome (ACS) is a clinical condition with a relative or absolute reduction in blood supply to the myocardium. Definition may be drawn on physical, electrophysiological, biochemical, radiological, and histopathological levels. Most frequent and, therefore, clinically most relevant types consist of atherosclerotic steno-occlusion of epicardial arteries with or without elevation of the ST segment on ECG (ST-elevation ACS or non-ST-elevation ACS, STE-ACS, or NSTE-ACS) [1].

ACS remains a leading cause of morbidity and mortality worldwide and a frequent cause of hospital admission and urgent cardiovascular care [1,2,3]. Advances in diagnostic modalities, pharmacotherapy, and interventional cardiology, including the development of novel antiplatelet agents, improved stent technologies, and refined imaging techniques, have significantly improved the management and outcomes of patients with ACS. Despite these advances, coronary artery bypass grafting (CABG) continues to play a role, particularly in patients with complex coronary anatomy, such as left main or multivessel disease, or when percutaneous coronary intervention is incomplete, unsuccessful, or otherwise relatively contraindicated. CABG in the context of acute myocardial infarction presents distinct challenges. Operative risk is affected not only by haemodynamic instability, extent of myocardial damage, and comorbidities, but also by the impact of potent antiplatelet and anticoagulant therapies, which are routinely administered as part of early management of ACS.

Nevertheless, the optimal timing of CABG in patients with ACS remains a subject of debate, particularly following myocardial infarction. Determining the appropriate timing requires careful balancing of the risks associated with early surgical intervention against the potential benefits of timely and complete revascularization [4].

In addition to timing, several clinical variables have been consistently identified as predictors of adverse outcomes following CABG. These include cardiogenic shock, reduced left ventricular systolic function, compromised pulmonary function, and renal insufficiency, all of which have been shown to independently increase perioperative and long-term risk [2,5,6].

This review aims to provide a comprehensive overview of recent developments in the management of ACS, with a particular focus on the role of CABG. It emphasizes the latest evidence concerning the optimal surgical timing, perioperative considerations, and risk stratification strategies. By synthesizing current guidelines and emerging data, we aimed to elucidate the evolving landscape of surgical intervention in ACS and support clinical decision-making in this high-stakes context.

## 2. Methods

### 2.1. Objectives

Our aim was to gather information from the last 25 years about timing considerations regarding CABG in ACS with emphasis on hard endpoint (MACCEs) outcomes according to the timing of the surgery. Such a relatively long period had to be targeted due to the small quantity and notable heterogeneity of the available literature.

### 2.2. Search Strategy

In this review, 25 studies were identified from an initial pool of 2302 records following a predefined screening process.

A comprehensive search of the Google Scholar (Alphabet Inc., Mountain View, CA, USA), PubMed (National Library of Medicine, Bethesda, MD, USA) Scopus (Elsevier B.V, Amsterdam, The Netherlands), and Web of Science (Clarivate PLC, London, UK) databases was conducted for studies published between January 2000 and February 2025. The search was guided by the following criteria: “(Myocardial infarction OR angina, Unstable OR Acute coronary syndrome) AND (Cardiac surgical procedures OR Myocardial revascularization OR Coronary artery bypass) AND (Disease-free survival OR Survival analysis OR Survival OR Hospital mortality OR Mortality OR Postoperative complications) AND (Timing).”

### 2.3. Study Selection Criteria

Titles and abstracts were evaluated according to established inclusion and exclusion criteria. Only articles published in peer-reviewed journals and accessible in a full-text PDF format were considered. After collegial discussion, the most significant papers were selected to serve as the foundation for the current review. After initial screening, the full texts of the selected studies were retrieved for a second round of eligibility assessment. Additionally, the reference lists of articles in these selected studies were examined to identify any pertinent articles that the original search strategy might have missed. Meta-analyses were solely used as background information during the work-up.

### 2.4. Limitations in Selection

This review was limited to English-language, peer-reviewed publications indexed in Google Scholar, PubMed, Scopus, and Web of Science.

The majority of the included studies were retrospective in design, including registry- and database-based analyses. As the primary focus of this review was the impact of surgical timing, substantial variability in timing definitions, endpoint composition, and patient selection precluded meaningful quantitative synthesis. These methodological limitations were, therefore, considered when interpreting the findings.

## 3. Results

**Figure 1:**
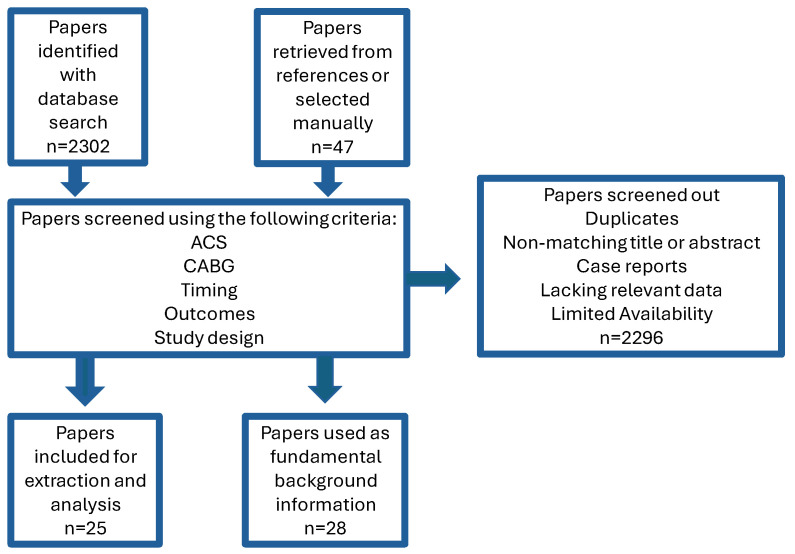
Selection of included literature (ACS: acute coronary syndrome, CABG: coronary artery bypass grafting).

Upon completing the above-described screening process, a total of 2302 studies were identified as potentially relevant, and finally, 25 of them were selected for detailed analysis, with a further 28 sources used as fundamental support (Figure 1). Among the included studies, 17 primarily focused on the impact of surgical timing, whereas 8 compared outcomes between CABG and PCI (Table 1 and Table 2). Fifteen of the articles consisted of database/registry/reviews or multicentre retrospective cohort analyses, eight were single-centre retrospective observations, and two were single-centre prospective studies.

Timing cohorts were different among various observations, and the most frequently used threshold values were 24, 48, 72 h, and 7 days. The observed endpoints were in-hospital mortality and major adverse cardiac and cerebral events (MACCEs) in most series among the timing-focused investigations and long-term survival among the intervention modality-focused studies. MACCEs are variably defined across studies, most commonly as a three-component composite endpoint (cardio- or cerebrovascular death, stroke, and myocardial infarction) or as a five-component endpoint that additionally includes hospitalization for heart failure or unstable angina, often incorporating repeat revascularization [7]. When bypass surgery was performed early—within 48 h—most authors [8,9,10,11,12,13,14,15] documented higher early mortality in the STE-ACS population. This paradoxically seems to contradict the “Golden Hour” concept of PCI in STE-ACS [5]. The NSTE-ACS patient group is less homogenous than the STE-ACS cohort, making it more difficult to determine a clearly ideal time for surgery, as numerous—potentially antagonistically influencing—factors need to be considered. Particularly highly (>100× upper level of normal) elevated troponin (Tn) levels show a strong correlation with inferior results in case of earlier surgeries [16,17]. Most series failing to demonstrate differences along the post-NSTE-ACS timeline for mortality and Major Adverse Cardiac and Cerebrovascular Events (MACCEs) emphasize, however, that similar results mask a more comorbid population among the lengthier preoperative stabilization cohort [9,18,19,20]. As a result, published studies do not show a trend similar to STE-ACS cases regarding the optimal timing of surgery. On the other hand, the actual risk profile of operated patients seemed more severe among non-stabilized patients.

### 3.1. Contemporary Role of CABG in the Management of ACS

When both revascularization strategies are technically feasible, PCI offers a clear short-term advantage in terms of procedural timing and length of hospital stay, particularly in time-sensitive clinical scenarios [1,4,5,21]. However, approximately 40% of ACS presentations occur in the setting of underlying multivessel coronary artery disease (MV-CAD) [22].

The advantages of CABG involve overall survival benefit, a chance for a more complete revascularisation, and lower long-term MACCE statistics [22]. These are more pronounced for complex multivessel coronary artery disease (CAD) in diabetic patients [23], with particular long-term gains among patients under 70 years of age or with reduced LVEF [24].

The utilization of CABG in NSTE-ACS has declined over the past two decades. Although no single explanatory factor has been identified, the continuous evolution and expanding applicability of PCI techniques likely play a substantial role [25].

### 3.2. Risk Stratification and Decision-Making Frameworks

CABG is preferred over PCI for diabetic patients in elective and urgent settings alike, as this cohort is known to have a reduced response to various platelet aggregation inhibitors (PAIs) and a less favourable long-term patency among patients undergoing PCI [1,2,5,26]. DM is also a risk factor for wound healing complications (and, as a consequence, inferior survival) among surgical patients (with a strong emphasis on TIIDM) [27]. However, recent data suggest that euglycaemic DM patients do not present the latter risk [24].

The challenges of indicating an open surgical intervention are complex, as the probable absolute surgical risk of a certain case and the relative advantage compared to the alternatives may diverge in the majority of cases. Patients with high overall surgical risk may benefit more from urgent open surgery compared to their peers, and even more so than some of those who would be better candidates for the procedure itself [4].

A number of methods have been developed to assess the urgency of action in ACS. Regarding efficacy and on-the-spot applicability, the Global Registry of Acute Coronary Events (GRACE) scoring system [3] has become the most widespread of all.

The anatomical complexity and procedural risk of PCI are commonly assessed using the Synergy Between PCI With Taxus and Cardiac Surgery (SYNTAX) score and its updated SYNTAX II model, with additional adaptations available for culprit-lesion assessment [28,29].

**Figure 2:**
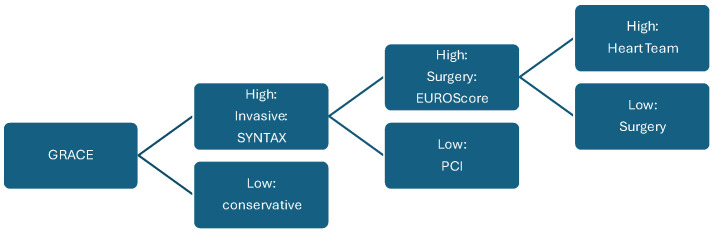
Key decision points of the ACS treatment process: GRACE score defines need for invasive measures, SYNTAX score defines anatomical complexity and procedural risk of a percutaneous intervention, and EUROScore judges perioperative risk of an open surgery [3,28,30].

Calculation of the periprocedural risk of CABG is widely performed by means of EuroScore II or STS Score assessment [30,31], both of which are considered equivalent in terms of predictive value [32]. Figure 2 summarizes the complementary roles of these scoring systems within a structured decision-making framework for ACS management [3,28,30].

### 3.3. Combined Scoring Approaches

Although these metrics have been developed and refined to assess peri- and early postprocedural hazard levels, both Syntax and EuroScore values also offer prognosis for long-term survival. The Syntax score does not predict outcomes after surgery and Euroscore has not been validated in the PCI population. EuroSCORE II generally outperforms SYNTAX II in predicting long-term mortality and major adverse cardiac and cerebrovascular events, whereas SYNTAX II is more focused on procedural outcomes and anatomical complexity, making it useful for planning PCI [33,34,35].

**Figure 3:**
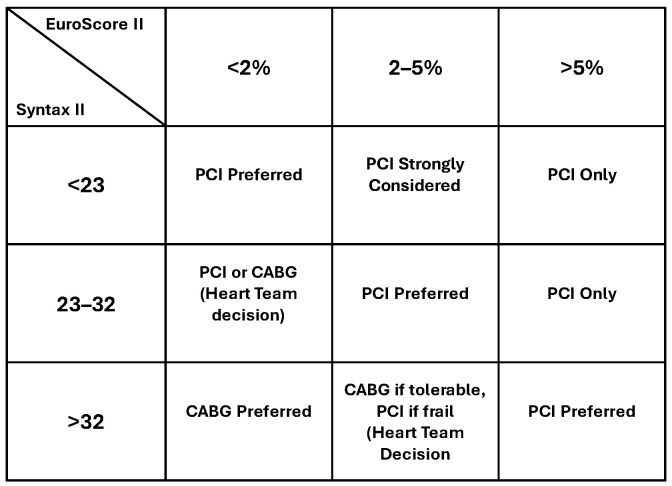
Decision-making matrix in ACS combining risk profiles from SYNTAX and EUROScore calculations (CABG: coronary artery bypass grafting, PCI: percutaneous coronary intervention) [1,28,30,36].

Several studies suggest that combining EuroSCORE II with SYNTAX score improves the overall predictive accuracy for outcomes in patients undergoing PCI or CABG [1,36]. This combined approach leverages the strengths of both scores, providing a more comprehensive risk assessment. A matrix of the possible combinations of SYNTAX and EUROScore risk profiles is summarized in Figure 3, highlighting the following areas of straightforward decisions or complex discussions: cases of high surgical risk or “neutral” risk profile favour PCI, whereas complex pathomorphology with or without severe comorbidities emphasizes the potential of open surgery.

### 3.4. High-Risk Subgroups and Special Clinical Scenarios

A database review on cases of ACS complicated by cardiogenic shock highlighted that patients receiving CABG yielded an in-hospital mortality rate of 19%, whereas the PCI arm had a mortality risk of 27% with salvage indications and the use of pre-, intra-, or postoperative mechanical circulatory support as the most prominent risk factors among CABG patients [37].

A systematic review of 36 studies involving 4321 patients with mechanical complications of ACS found no significant difference in early (32.6%) or late mortality (40.0%) between patients who underwent concomitant CABG and those who did not. This suggests that CABG does not significantly impact overall survival rates in the short or long term for most mechanical complications of ACS. However, in cases of papillary muscle rupture, concomitant CABG was associated with significantly lower long-term mortality (RR 0.42; *p* = 0.001), indicating a potential benefit of CABG in specific types of mechanical complications [38].

Although the question of repeat revascularisation is a complex issue—as a repeat PCI in terms of duration of recovery and added procedural risk is definitely less demanding than a redo CABG—most studies consider “unplanned” (not foreseen at the time of the initial revascularisation, but not necessarily urgent) or “acute” re-interventions equally significant events as surgical reoperations [24].

Despite robust evidence supporting the long-term survival advantage of CABG, the routine use of multiple arterial grafting remains limited in contemporary practice. A small proportion of surgeons perform the majority of multi-arterial reconstructions, suggesting that further improvements in surgical outcomes may be achievable through broader adoption of established techniques [39].

**Table 1:** Core findings of studies involving timing factors included in the review (ACS: acute coronary syndrome, CABG: coronary artery bypass grafting, cTn: conventional troponin, d: day, DM: diabetes mellitus, LT: long-term, LVEF: left ventricular ejection fraction, MACCEs: major adverse cardiac and cerebrovascular events, NSTE-ACS: non-ST-elevation acute coronary syndrome, ST: short-term, STE-ACS: ST-elevation acute coronary syndrome, TIDM: type I diabetes mellitus, TIIDM: type II diabetes mellitus, Tn: troponin).

Study	Study Design	Population	Timing Considerations	Endpoints	Key Findings	Limitations
Acharya et al., 2016 [9]	registry	CABG < 7 days after ACS	salvage/emergent/urgent	MACCE	earlier cases—worse risk profile—worse outcomes	CABG indication based on clinical routine duration of shock not available MCS use influenced by local and individual practice
Benedetto et al., 2022 [16]	multi-centre retrospective cohort	CABG after NSTE-ACS	days from Tn peak to surgery	30 d/LT mortality	if peak Tn is <100× normal, no delay needed; if >100× normal, then 5–10 days seems beneficial	retrospective lacking data on: anatomical complexity, surgical technique, completeness of revascularisation, medication, cause of death
da Fonseca et al., 2018 [40]	single-centre retrospective	awaiting elective CABG	duration of waiting status	MACCE	waiting over 16 weeks yields a risk in itself—significant additional risk factor is reduced LVEF (<45%)	lacking data on: drug adherence, timing of surgery, patients with end points treated at another provider
Hadaya et al., 2022 [41]	registry	CABG after ACS	0, 1–3, 4–7, >7 d	MACCE	worst results recorded on day 0 and in >7 groups; day 1–3 and 4–7 were better, with day 1–3 presenting lower costs	retrospective time measured in days lacking data on: laboratory values, risk scores, antiplatelet drug use, LVEF
Huenges et al., 2024 [27]	single-centre retrospective	CABG < 48 h ACS among TIDM and TIIDM	<48 h	MACCE, LT survival	TIIDM showed worst risk profile, short- and long-term mortality followed by TIDM and non-diabetic	ongoing registry with changing clinical protocols selection bias
Kim et al., 2024 [14]	database review	CABG after <1 y ACS	<1, 1–2, 3–7, 7–21, >21 d	MACCE, survival	worst results 24–48 h	selection bias no STE-ACS vs NSTE-ACS distinction no randomisation of surgical methods
Lee et al., 2001 [12]	multi-centre retrospective cohort	CABG in NSTE.ACS and STE-ACS	<6 h, 6 h–1 d, 1–7 d, >7 d	ST survival	earlier surgery yields inferior outcomes; difference is lesser among NSTE-ACS	retrospective no data on timing protocol
Lee et al., 2003 [13]	multi-centre retrospective cohort	CABG in STE-ACS	<6 h, 6 h–1 d, 1–3 d, 4–7 d, 7–14 d, >14 d	ST survival	surgery < 3 d yields inferior outcomes	retrospective varying local protocols
Lemaire et al., 2020 [15]	database review	CABG in STE-ACS	1 d, 2–3 d, 4–7 d	ST survival, perioperative complications	1 d group had significantly inferior results	retrospective lacking data on: type of surgery, cause of timing
Liakopoulos et al., 2019 [42]	database review	NSTE-ACS and STE ACS with CS and CABG	<24 h, >24 h	MACCE, ST mortality	STE-ACS worse than NSTE-ACS; effect of timing is ns	lacking data on: presurgical treatment, completeness of revascularisation, long-term clinical data institutional bias
Parikh et al., 2010 [20]	database review	CABG in NSTE-ACS	<48 h, >48 h	ST MACCE, mortality	more risk factors in the late group, ns results	retrospective bias: survival and selection locally variable protocols low overall GRACE scores
Patlolla et al., 2024 [43]	single-centre retrospective	CABG in NSTE.ACS and STE-ACS	<24 h, 1–7 d, >7 d	ST mortality, MACCE	inferior outcomes > 7 d, especially in elderly people, NSTE-ACS, DM	retrospective lacking data on: pre-center treatment, cause of death, MACCE selection bias
Rojas et al., 2019 [18]	single-centre prospective observational	CABG in NSTE-ACS	<72 h, >72 h	6 mo mortality, MACCE	more risk factors in the early group, ns results	retrospective selection bias favoring late surgeries only short-term follow-up
Thielmann et al., 2021 [19]	database review	CABG after PCI (culprit with further indications, failed or abandoned)	<24 h, >24 h	ST mortality, MACCE	high-risk subpopulation among CABG due to baseline characteristics	norandomized incompleteness of patient and pre-surgical treatment data possible treatment bias
Thielmann et al., 2006 [17]	single-centre prospective	CABG in STE-ACS and NSTE-ACS	<24 h	ST mortality, MACCE	preoperative cTnI was a strong predictor of survival in both groups	only short-term follow-up single center
Voisine et al., 2005 [10]	single-centre retrospective	CABG after ACS	<6 h, 6–24 h, 1–7 d, 8–30 d, >30 d	ST mortality	inferior outcomes < 7 d—especially among historically earlier surgeries	retrospective no NSTE-ACS and STE-ACS distinction cause of urgency is partly missing
Weiss et al., 2008 [11]	database review	CABG after ACS	<24 h, 24–48 h, 48–72 h, >3 d	ST mortality	inferior outcomes < 3 d	retrospective possible selection bias favoring late surgeries missing clinical data includeing LVEF, NYHA

**Table 2:** Studies involving percutaneous vs. open surgical aspects included in the review (ACS: acute coronary syndrome, CABG: coronary artery bypass grafting, LT: long-term, MACCEs: major adverse cardiac and cerebrovascular events, NSTE-ACS: non-ST-elevation acute coronary syndrome, PCI: percutaneous coronary intervention, ST: short-term, STE-ACS: ST-elevation acute coronary syndrome).

Study	Study Design	Population	Timing Considerations	Endpoints	Key Findings	Limitations
Elbadawi et al., 2019 [44]	multi-centre retrospective cohort	mechanical complication after ACS	n. a.	in-hospital mortality	rate does not decrease, outcomes do not improve vastly, surgery still yields benefits	retrospective lack of data on: laboratory findings, LVEF, pre-treatment delay
Ezad et al., 2018 [45]	single-centre retrospective	urgent CABG after PCI	n. a.	in-hospital mortality	PCI complications are rare but present and urgency is mandatory despite the high surgical risk	retrospective single center low caseload no statistical end points reviewed
Fukui et al., 2014 [33]	single-centre retrospective	undergoing isolated CABG	n. a.	MACCE	both Euroscore II and SYNTAX II judge early risk appropriately; for long-term prognosis, Euroscore II fits	retrospective single center limited number of patients
Huckaby et al., 2020 [46]	single-centre retrospective	NSTE-ACS multivessel PCI or CAB	unknown	MACCE, LT survival	lower mortality and MACCEs among CABG group	retrospective outpatients not included lack of data on: anatomical complexity, LVEF
Mehaffey et al., 2023 [47]	database review	age > 65 y ACS CABG or multivessel PCI	unknown	MACCE, LT survival	higher stroke and rate, cost, longer hospitalization but lower mortality in the shorter term, lower mortality and MACCEs in the longer term among CABG group	retrospective lack of data on: anatomical complexity, heart team involved, patient preference
Mehta et al., 2008 [37]	database review	ACS CABG	n. a.	ST mortality	mortality not prohibitive	retrospective selection bias (referral)
Omerovic et al., 2024 [24]	database review	multivessel NSTE-ACS	n. a.	mortality ST, LT, MACCE	stroke ns, other MACCEs and mortality prefer CABG	retrospective evolving treatment during data collection period lack of data on: MCS use, graft and stent type, presence of PAD, CTO, completeness of revascularisation, adherence to medication
Paparella et al., 2010 [48]	single-centre retrospective	CABG < 21 d after ACS	n. a.	ST, LT mortality, MACCE	praeop. tropI > 0.15 worse outcomes	retrospective lack of consistent postoperative imaging data lack of initial troponin values

## 4. Discussion

Today, the role of CABG in ACS reflects a nuanced, evidence-based balance between the timing, risk of bleeding, and extent of coronary disease. While PCI remains first-line in most ACS cases due to its shorter door-to-needle and door-to-balloon times [4], CABG retains a vital role in the modern era—often as a complementary or rescue strategy, especially in patients with anatomy or comorbidities that confer a high risk of PCI.

## 5. Timing of CABG in STE-ACS and NSTE-ACS

The optimal timing of CABG in patients with ACS depends on the symptoms, biochemical findings, coronary anatomy, and haemodynamic stability. Decisions on timing should be discussed along with the choice of revascularisation method, as a fundamental advantage of PCI in comparison with CABG is its shorter procedural time [4].

### 5.1. Timing of CABG in STE-ACS

Primary PCI of the culprit lesion remains the gold standard treatment for acute STE-ACS. However, when PCI is not feasible, unsuccessful, or contraindicated (e.g., failed PCI, left main or multivessel disease with complex lesions or anatomy), emergent or urgent CABG may be considered [28].

Current guidelines recommend CABG immediately (within hours to 24–48 h) in STE-ACS patients with haemodynamic instability refractory to medical or device-based therapy or in the presence of mechanical complications such as ventricular septal rupture, acute mitral regurgitation, or free wall rupture [2,5,28,37].

Several observational studies indicate that delaying CABG until initial clinical stabilization—typically 3–7 days after STE-ACS—may reduce perioperative bleeding risk and early mortality, particularly in patients exposed to potent dual antiplatelet therapy [15].

While urgent open surgery in STE-ACS is the worst prognostic cohort, treatment of mechanical (~1/400 STEMI, 42% mortality) or procedural (~1/1000 PCI) complications (dissection, occlusion, or perforation of coronary branches) is responsible for a considerable proportion of the poor outcomes and is not necessarily distinguished on follow-up from other indication groups of immediate CABG procedures [8,38,44].

### 5.2. Timing of CABG in NSTE-ACS

Urgent CABG (within 24–48 h): Recommended for patients with high-risk features, left main disease, complex lesions, or severe triple-vessel CAD with ongoing ischemia despite optimal medical therapy [49].

Delayed CABG (3–7 days): Preferred when initial medical therapy allows stabilization, reducing perioperative bleeding risks due to potent PAI withdrawal [50].

Elective CABG (>7 days): Considered in stable patients after full risk assessment, optimal medical therapy, and multidisciplinary discussion [15].

An obvious risk in the case of elective CABG is the waiting list mortality, estimated at 5.3/100 patient-years, aggravated among patients with HFrEF (LVEF < 45%) [40,51].

Higher levels of peak Tn (>100× upper normal level) are associated with an increased risk of mortality in the case of early CABG among NSTE-ACS patients, whereas in the case of moderately elevated Tn levels (<100× upper normal level), the importance of timing does not seem to be critical [16,52].

An observational series found no differences in 30-day survival between groups being operated over or within 72 h after symptom onset, although patient characteristics were less favourable in the early group. Need for mechanical circulatory support was more frequent in this group as well [18].

### 5.3. Interpretation and Clinical Implications

Importantly, the observation that patients undergoing earlier CABG tend to have worse outcomes should not be interpreted as evidence against expedited surgery per se. Rather, this finding likely reflects confounding by indication, as patients referred for early or urgent CABG are frequently those with greater clinical instability or more severe presentations. Consequently, this selection bias may obscure or attenuate a potential beneficial effect of timely surgical revascularization. This phenomenon is conceptually similar to what is observed in other high-risk scenarios, such as cardiac arrest, where earlier intervention is often applied to the sickest patients.

## 6. Knowledge Gaps and Future Directions

Prognostic Models and Selection Algorithms Specific to ACS: A clinically validated and widely accepted decision-making tool is lacking for urgent CABG in ACS patients—one that accounts for haemodynamic status, ACS subtype, comorbidities, biomarkers, mechanical circulatory support (MCS), and antiplatelet therapy [53].

Shifting from Standard Delay to Patient-Centred Strategy: There is limited evidence to guide individualized timing decisions that consider haemodynamic trajectories, the use of contemporary MCS strategies, real-time stabilization indicators (e.g., end-organ perfusion), and current antiplatelet therapy across different ACS subtypes [54,55].

Optimizing the Use of Mechanical Circulatory Support in Urgent CABG: Mechanistic and outcome-driven analyses of MCS in urgent CABG are scarce, particularly with regard to strategy comparisons such as “bridge-to-stabilization” versus “bridge-to-surgery.” This highlights the need for prospective, registry-integrated studies to delineate the clinical course, indications, and outcomes associated with MCS before, during, and after surgery. While previous studies have failed to support the efficacy of IABP use [56], ECMO and Impella devices have been increasingly applied as a bridge to CABG in unstable patients with severe left ventricular dysfunction, VSR, or cardiogenic shock [57].

Urgency-stratified data on post-myocardial infarction mechanical complications treated with concomitant CABG remain limited. Dedicated multi-centre registries focusing on ventricular septal rupture or papillary muscle rupture—capturing timing from infarction, surgical techniques, and MCS utilization—are urgently needed to clarify optimal management strategies [38].

Long-Term Outcomes: Evidence regarding long-term outcomes beyond five years following urgent CABG in ACS remains sparse, particularly with respect to survival, MACCE incidence, graft patency, and repeat revascularization rates.

Perioperative Antiplatelet Management: In the case of elective or urgent surgeries, ticagrelor and prasugrel should be stopped 3–5 days before CABG to minimize bleeding risk, whereas clopidogrel requires 5–7 days of discontinuation [5,58]. Currently no evidence supports ASA withdrawal unless a condition with high bleeding risk is present [58,59]. Efforts with more or less convincing results have been made to present a predictive model for timely (first medical contact) distinction among NSTE-ACS patients based on the probability of urgent CABG indication and the benefit of avoiding the routine DAPT loading [60]. High bleeding risk may be another factor shifting patients towards the less invasive PCI when time is scarce for thorough preprocedural bleeding control [61].

## 7. Conclusions

Despite continuous technical advancements and substantially reduced door-to-needle and door-to-balloon times that have established PCI as the first-line strategy in acute settings, CABG retains an important role in selected cases. Anatomical and pathomorphological challenges [28] and, to a lesser extent, long-term survival advantages [24] continue to justify CABG when the heart team, including cardiac surgeons and interventional cardiologists, judges that the cumulative mortality and morbidity associated with acute PCI followed by staged PCIs or delayed CABG would exceed that of primary complete revascularization with acute CABG. Typical scenarios include heavily calcified trifurcating left main disease and multiple complex proximal lesions with the absence of a clearly identifiable culprit lesion.

An inherent and complex bias influencing indication arises from the observation that patients who are expected to earn greater benefit from salvage, emergent, or urgent CABG—as compared to PCI—often exhibit a higher absolute surgical risk profile. Conversely, those with a more favourable surgical risk are typically better candidates for PCI as well, pushing less “desirable” cases to the surgical pool [33].

A further double collider bias of treatment timing according to the severity of cardiac pathology (earlier intervention) and comorbidities (lengthier, nuanced preparation) is an inherent aspect of this population. As these barriers are closely linked with firm ethical boundaries, chances seem to be limited for further clarification.

While STE-ACS patients with mechanical complications or failed PCI may require urgent surgery even in a considerably high-risk setting, frail NSTE-ACS patients benefit from delayed CABG to optimize outcomes [11,12,16].

A less thoroughly elaborated question is whether the higher risk of urgent CABG procedures still prevails after the omission of cases indicated upon mechanical and procedural complications, which may require further studies. Current data converges to the idea that postponing CABG in an ACS setting in general might not yield the benefit of a lesser risk profile [38].

## 8. Limitations

The available literature addressing the timing and modality of revascularization in acute coronary syndromes is substantially affected by several sources of bias. Most studies are observational in nature and are, therefore, highly susceptible to confounding by indication, as patients selected for early or urgent coronary artery bypass grafting often differ systematically from those managed with delayed surgery or percutaneous coronary intervention. In addition, time-dependent bias represents a major methodological concern, since treatment allocation is intrinsically linked to clinical stability, evolving risk profiles, and survival to the time of intervention [62]. Accordingly, the findings summarized in this review should be interpreted as descriptive associations rather than causal inferences. Apparent differences in outcomes between revascularization strategies or timing categories may reflect underlying patient selection, disease severity, or logistical factors rather than true comparative effectiveness. Given the marked heterogeneity of study designs, patient populations, and endpoints, formal quantitative synthesis was not feasible. These limitations underscore the need for cautious interpretation of the existing evidence and highlight the importance of well-designed prospective studies specifically addressing timing-related questions in acute coronary syndromes.

## Data Availability

No new data were created or analyzed in this study.

## Abbreviations

ACS	Acute Coronary Syndrome
AI	Artificial Intelligence
CABG	Coronary Artery Bypass Grafting
DAPT	Dual Antiplatelet Therapy
DM	Diabetes Mellitus
ECMO	Extracorporeal Membrane Oxygenator
GRACE	Global Registry of Acute Coronary Events
IABP	Intra-Aortic Balloon Pump
LT	Long-term
MACCE	Major Adverse Cardiac and Cerebrovascular Events
MCS	Mechanical Circulatory Support
NSTE-ACS	Non-ST-elevation Acute Coronary Syndrome
PAI	Platelet Aggregation Inhibitor
PCI	Percutaneous Coronary Intervention
RCT	Randomized Controlled Trial
ST	Short-term
STE-ACS	ST-elevation Acute-Coronary Syndrome
SYNTAX	Synergy Between PCI with Taxus and Cardiac Surgery
TIDM	Diabetes Mellitus Type I
TIIDM	Diabetes Mellitus Type II
VSR	Ventricular Septal Rupture
